# Development and evaluation of a CRISPR/Cas12a-based diagnostic test for rapid detection and genotyping of HR-HPV in clinical specimens

**DOI:** 10.1128/spectrum.02253-24

**Published:** 2024-11-21

**Authors:** Lijuan Yin, Ziqian Zhao, Chunhua Wang, Caihong Zhou, Xiuzhen Wu, Baoxue Gao, Liangyuan Wang, Shuli Man, Xinkuan Cheng, Qiankun Wu, Siqi Hu, Hongxia Fan, Long Ma, Hui Xing, Liang Shen

**Affiliations:** 1 State Key Laboratory of Food Nutrition and Safety, Key Laboratory of Industrial Microbiology, Ministry of Education, Tianjin Key Laboratory of Industry Microbiology, National and Local United Engineering Lab of Metabolic Control Fermentation Technology, China International Science and Technology Cooperation Base of Food Nutrition/Safety and Medicinal Chemistry, College of Biotechnology, Tianjin University of Science & Technology, Tianjin, China; 2 Department of Clinical Laboratory, Xiangyang Central Hospital, Affiliated Hospital of Hubei University of Arts and Science, Xiangyang, Hubei Province, China; 3 Department of Clinical Laboratory, Xiangyang No.1 People's Hospital, Hubei Universitly of Medicine, Xiangyang, China; 4 Dynamiker Sub-Center of Beijing Key Laboratory for Mechanisms Research and Precision Diagnosis of Invasive Fungal Disease, Tianjin, China; 5 Academy of National Food and Strategic Reserves Administration, Beijing, China; 6 Institute of Pediatrics, Faculty of Pediatrics, The Seventh Medical Center of Chinese PLA General Hospital, Beijing, China; 7 Department of Pathogen Biology, School of Basic Medical Sciences, Tianjin Medical University, Tianjin, China; Shandong First Medical University, Jinan, Shandong, China

**Keywords:** CRISPR/Cas12a, multiplex, detection, HR-HPV, genotyping, clinical samples

## Abstract

Persistent infection with high-risk human papillomavirus (HR-HPV) is the principal etiological factor of cervical cancer. Considering the gradual progression of cervical cancer, the early, rapid, sensitive, and specific identification of HPV, particularly HR-HPV types, is crucial in halting the advancement of the illness. Here, we established a rapid, highly sensitive, and specific HR-HPV detection platform, leveraging the CRISPR/Cas12a assay in conjunction with multienzyme isothermal rapid amplification. Our platform enables the detection and genotyping of 14 types of HR-HPV by using type-specific crRNAs. The outcomes of the detection can be interpreted either through a fluorescence reader or visually. Furthermore, we achieved one-tube multiplex detection of 14 HR-HPV types through the use of multiple amplifications and a crRNA pool. The detection sensitivity of this method is 2 copies/μL with no cross-reactivity, and the results can be obtained within 30 minutes. This method exhibited 100% clinical sensitivity and 100% clinical specificity when applied to 258 clinical specimens. Based on these findings, our CRISPR/Cas-based HR-HPV detection platform holds promise as a novel clinical detection tool, offering a visually intuitive and expedited alternative to existing HPV infection diagnostics and providing fresh perspectives for clinical cervical cancer screening.

**IMPORTANCE:**

This study developed a novel high-risk human papillomavirus (HR-HPV) detection platform based on CRISPR/Cas12a technology. This platform not only enables the rapid, highly sensitive, and specific detection and genotyping of 14 types of HR-HPV but also achieves single-tube multiplex detection of 14 HR-HPV types through ingenious design. The outcomes of the detection can be interpreted either through a fluorescence reader or visually. To the best of our knowledge, this is the first paper to utilize CRISPR/Cas diagnostic technology for the simultaneous detection of 14 types of HPV and to evaluate its feasibility in clinical sample detection using a large number of clinical samples. We hope that this work will facilitate the rapid and accurate detection of HPV and promote the broader application of CRISPR/Cas diagnostic technology.

## INTRODUCTION

Persistent high-risk human papillomavirus (HR-HPV) infection is the main risk factor for the occurrence of cervical cancer, which seriously threatens women’s health (1–3). The World Health Organization (WHO) and the International Agency for Research on Cancer (IARC) estimated that there were around 604,127 new cervical cancer cases worldwide in 2020, with a mortality rate of 7.2 deaths per 100,000 woman-years (4). Most of the new cases and deaths were from low- and middle-income countries. There are 14 types of HR-HPV (types 16, 18, 31, 33, 35, 39, 45, 51, 52, 56, 58, 59, 66, and 68) that are closely linked to precursor lesions and carcinomas, which are commonly found in over 90% of cervical cancer patients (5, 6). Among them, types 16 and 18 collectively contribute to almost 70% of high-grade cervical pre-cancers (7). Therefore, the early detection and genotyping of HR-HPV using a rapid and simple method are crucial for the screening, diagnosis, and treatment of cervical cancer.

HPV DNA testing has been recommended by the WHO as the preferred screening approach for cervical cancer prevention (8). At present, various HPV DNA determination and genotyping approaches have been developed, including real-time quantitative PCR (qPCR), digital PCR, and DNA sequencing (9–11). Real-time quantitative PCR is the most frequently employed technique for HPV DNA detection due to its obvious advantages such as relatively high sensitivity and specificity (12). For example, the Cobas HPV test and Cepheid Xpert HPV assay, which are designed based on qPCR, have been widely used for the screening of the 14 types of HR-HPV. However, these methods still face some limitations such as complicated detection processes, costly equipment, poor reliability for low-concentration samples, difficulty in performing multiple amplifications, and infeasibility for field application (13, 14). These limitations restrict its application in settings with limited facilities or point-of-care testing. Digital PCR offers higher sensitivity than qPCR and allows for absolute quantification. However, it also has shortcomings in rapid, simple, and multiplex detection (15). DNA sequencing, including next-generation DNA sequencing and nanopore DNA sequencing, provides detailed sequence information and enables the detection of various types of pathogens simultaneously. Nonetheless, it is still costly and time-consuming (16). The fast, inexpensive, multiplex genotyping and simple detection requirements remain unmet. Therefore, there is a requirement to develop novel techniques for detecting HR-HPV DNA with straightforward operation, high sensitivity and specificity, multiplexed capacity, and field suitability.

The clustered regularly interspaced short palindromic repeats (CRISPR)-Cas (CRISPR-associated proteins) is an adaptive immune system in bacteria and archaea. This system provides resistance against invading mobile genetic elements, such as phages and plasmids, owing to its adaptive nature (17). Apart from the particular recognition and cleavage of the target nucleic acid (*cis*-cleavage activity), certain Cas proteins, such as Cas12a and Cas13a, also exhibit *trans*-cleavage activity when binding to a specific target, leading to the indiscriminate cleavage of the single-stranded DNA (ssDNA) or RNA (18, 19). Based on this property, CRISPR/Cas systems have been developed as novel nucleic acid detection technologies (20). Various detection methods, such as SHERLOCK, HOLMES, and DETECTR, which utilize the high sensitivity and specificity of CRISPR-Cas proteins, have been employed to identify various pathogens, such as SARS-CoV-2 (21, 22), monkeypox virus (23), *Staphylococcus aureus* (24), and *Salmonella* (25), and to distinguish single-base differences (19, 26).

In this study, we developed a highly sensitive CRISPR/Cas12a-based fluorescence detection method for efficient identification of 14 types of HR-HPV. Our method successfully detected all 14 types of HR-HPV in a single tube through the utilization of multiple amplifications and a crRNA pool. Furthermore, we evaluated the test’s diagnostic performance for detecting HR-HPV in clinical samples, showing 100% clinical sensitivity and 100% clinical specificity. This method allows simple, rapid, sensitive, selective, multiplex, and genotyping detection of HR-HPV DNA in clinical samples. We believe that this method has the potential to be a clinical detection tool, providing a visually intuitive and expedited alternative to current HPV infection diagnostics. It has broad applications in diagnosing specific infections, yielding substantial health and clinical advantages, and presenting novel approaches for clinical cervical cancer screening.

## MATERIALS AND METHODS

### Materials

The HPV amplification primers and crRNAs used in this study were synthesized by Tsingke Biotech (Beijing, China), with detailed sequences provided in Table S1. Reporter ssDNA (5′-FAM-TTATT-BHQ1-3′) was synthesized by Tsingke Biotech (Beijing, China) and was labeled with a fluorophore (FAM) at the 5′ end and a quencher (BHQ1) at the 3′ end. The HPV plasmid was constructed by cloning the specific type of HPV L1 gene into the pUC57 vector using EcoRI and BamHI restriction sites. The purification of *Lachnospiraceae bacterium* ND2006 Cas12a protein (LbCas12a) and generation of crRNA were conducted as our previous work (25). T7 High Yield RNA Synthesis Kits (#E2050S) and Monarch RNA Cleanup Kits (#T2040L) were acquired from New England Biolabs (USA). Genomic DNA extraction kits were acquired from Odrei Biomedical Technology Co., Ltd. (Tianjin, China). Multienzyme isothermal rapid amplification (MIRA) kit was acquired from AMP-Future Biotech Co., Ltd. (Shandong, China). Chemical reagents utilized in this study were sourced from Sigma-Aldrich (USA) or Solarbio Science & Technology Co., Ltd. (Beijing, China). Isopropyl-β-D-thiogalactoside was sourced from Sigma-Aldrich (USA). Other chemical reagents were obtained from Solarbio Science & Technology Co., Ltd. (Beijing, China).

### Single-plexed MIRA assay

MIRA reactions were conducted using a commercially accessible kit in accordance with the instructions. Simply, the rehydration solution, consisting of 4 µL forward and reverse primers (10 µM), 29.4 µL rehydration buffer, 2 µL DNA template (with varying concentrations for different purposes), and 12.1 µL ddH_2_O, was added to the reaction pellet and thoroughly mixed through repetitive pipetting. Next, magnesium acetate (280 µM) was introduced to initiate the amplification process. The amplification process was carried out at 39℃ for 15 minutes. In the case of the negative control, the plasmid without the gene insert was used in the reaction.

### 2-, 5-, 12-, and 14-plexed MIRA assay

First, the reaction solution was prepared by transferring the rehydration buffer (29.4 µL) into the reaction pellet. Then, DNA templates and primers were added. For 2-plexed MIRA, DNA templates containing HPV-16 and HPV-18 L1 gene (2 fmol for each type) and 2 types of amplification primers (final concentrations: 200 nM) were included into the reaction solutions. For 5-plexed MIRA, DNA templates containing HPV (types 16, 18, 33, 52, and 58) L1 gene (2 fmol for each type) and 5 types of amplification primers (final concentrations: 200 nM) were added into the reaction solutions. For 12-plexed MIRA, DNA templates containing HPV (types 31, 33, 35, 39, 45, 51, 52, 56, 58, 59, 66, and 68), L1 gene (2 fmol for each type), and 12 types of amplification primers (final concentrations: 200 nM) were added into the reaction solutions. For 14-plexed MIRA, DNA templates containing all 14 HR-HPV subtypes L1 gene (2 fmol for each type) and 14 types of amplification primers (final concentrations: 200 nM) were added into the reaction solutions. After that, magnesium acetate (2.5 µL, 280 µM) and ddH_2_O were introduced. The final reaction volume for each setup was 50 µL. The amplification reaction proceeded at 42℃ for 15 minutes.

### Feasibility analysis of CRISPR/Cas12a-based detection

For CRISPR/Cas12a-based assay, the reaction mixture containing 100 nM Cas12a, 200 nM crRNA, 200 nM reporter ssDNA, 50 mM NaCl, 10 mM MgCl_2_, 10 mM Tris-HCl, 10 mM DTT, and different concentrations of MIRA product (2 µL) was prepared and added into a 96-well microplate. Then, the microplate was incubated in a qPCR detection system (QuantStudio 1 Plus Real-Time PCR Instrument, Thermo Fisher, USA) at 37 ℃ for 60 minutes, with fluorescence signals collected every 30 seconds (λex: 485 nm; λem: 535 nm). After the reaction, the fluorescent signals were observed and imaged under both ultraviolet (UV) light illuminators and blue LED lighting conditions using a MiniChemi Chemiluminescence Fluorescence Imaging Analysis System (SINSAGE, China).

### The optimization of the CRISPR/Cas12a-based detection

For the optimization of reaction temperature, the CRISPR/Cas12a-based detection assay was performed at various temperatures ranging from 37°C to 41°C for 30 minutes. The fluorescence signal and signal-to-background ratio were determined. To optimize the concentration of Cas12a protein, reaction mixtures were prepared with different concentrations of LbCas12a protein (50 nM, 100 nM, 200 nM, 400 nM, and 600 nM), along with 200 nM crRNA, 200 nM reporter ssDNA, 50 mM NaCl, 10 mM MgCl_2_, 10 mM Tris-HCl, 10 mM DTT, and a constant volume of MIRA product (2 µL). The reactions were conducted at 37°C for 30 minutes. The fluorescence signal and signal-to-background ratio were determined. For the optimization of DTT concentration, CRISPR/Cas12a-based reactions were carried out using buffers containing different concentrations of DTT (0 mM, 1 mM, 10 mM, and 20 mM). The cleavage activity of Cas12a was determined by monitoring the fluorescence signal and signal-to-background ratio.

### The sensitivity and specificity assay for HPV detection

For sensitivity assay, 2 µL of 10-fold serial dilutions of HPV16 or HPV18 plasmid DNA from 10^−2^ to 10^5^ copies/μL were amplified with single-plexed MIRA assay. The HPV16 and HPV18 plasmids were constructed by cloning the HPV16 and HPV18 L1 genes into pUC57 vector using EcoRI and BamHI restriction sites. The control group was set up using the empty vector pUC57 as the amplification template instead of HPV plasmid DNA. Then, 2 µL MIRA products were tested by CRISPR/Cas12a-based assay. For specificity assay, common microorganisms and human cells were tested, including low-risk HPV type 6 and type 11, *Candida krusei* (*C. krusei*), *Candida albicans* (*C. albicans*), *Candida parapsilosis* (*C. parapsilosis*), *Candida tropicalis* (*C. tropicalis*), *Escherichia coli* (*E. coli*), *Staphylococcus aureus* (*S. aureus*), group B *Streptococcus* (GBS), *Salmonellas*, SARS-CoV-2, influenza A virus (IAV), herpes simplex virus type (HSV), and Hela cells. Specifically, the same amount of genomic DNA from common microorganisms and human cells was amplified at 39℃ for 15 minutes with HPV16 or HPV18-specific primers. Subsequently, the amplification products were detected using the CRISPR/Cas12a assay.

### 14 × 14 matrix test

The target genes of 14 types of high-risk HPV were separately amplified according to the procedure of the single-plexed MIRA assay using type-specific primer pairs. An equal amount of 2 fmol of plasmids containing the HPV L1 gene was used as amplification templates. The amplicons were detected by the CRISPR/Cas12a assay. crRNAs targeting the specific sequences of each of the 14 HR-HPV types were synthesized. Each crRNA was tested against all 14 HPV amplicons to create a comprehensive matrix.

### Denaturing PAGE

A reaction containing 100 nM LbCas12a, 200 nM crRNA, 1 µM ssDNA, and 2 µL MIRA products was incubated in the CRISPR/Cas12a reaction buffer at 37°C for 60 minutes. After the incubation, the cleavage reaction was stopped by heating at 65°C for 10 minutes. Then, the cleavage products were analyzed with 20% denatured PAGE gel. The results were read using a fully automated DNA gel imager (BioRad Company, USA).

### Clinical sample detection

A total of 258 clinical cervical swab samples were obtained from the Xiangyang Central Hospital and endorsed by the ethics committee (2022-089), including 97 samples positive with HPV16, 23 samples positive with HPV18, 80 samples positive with other high-risk HPVs (non-16/18 HPV), and 58 negative samples from healthy individuals. Genomic DNA from the clinical cervical swabs was extracted using the commercially available nucleic acid extraction kit (Odrei Biomedical Technology Co., Ltd.). A total of 258 clinical samples were tested utilizing the Cobas HPV test (Roche Diagnostics, Basel, Switzerland) and CRISPR/Cas12a-based assay.

### Quantification and statistical analysis

The concentration of the plasmid containing HPV L1 gene was measured using a UV-Vis spectrophotometer at 260 nm. The copy number of HPV plasmid was calculated from the concentration of the DNA. The formula for calculating the DNA copy number is as follows: DNA copy number per microliter = [(6.02 × 10^23^) × (plasmid concentration, in nanograms per microliter) × 10^−9^]/[(fragment length, in base pairs) × 660]. The data gathered from three separate experiments were presented as the average values, with the variation represented as ± standard deviation (SD). The statistical significance was calculated using a Student’s *t*-test. A *P*-value less than 0.05 was regarded as statistically significant.

## RESULTS

### Development of MIRA-CRISPR/Cas12a method for HPV detection

**Fig 1:**
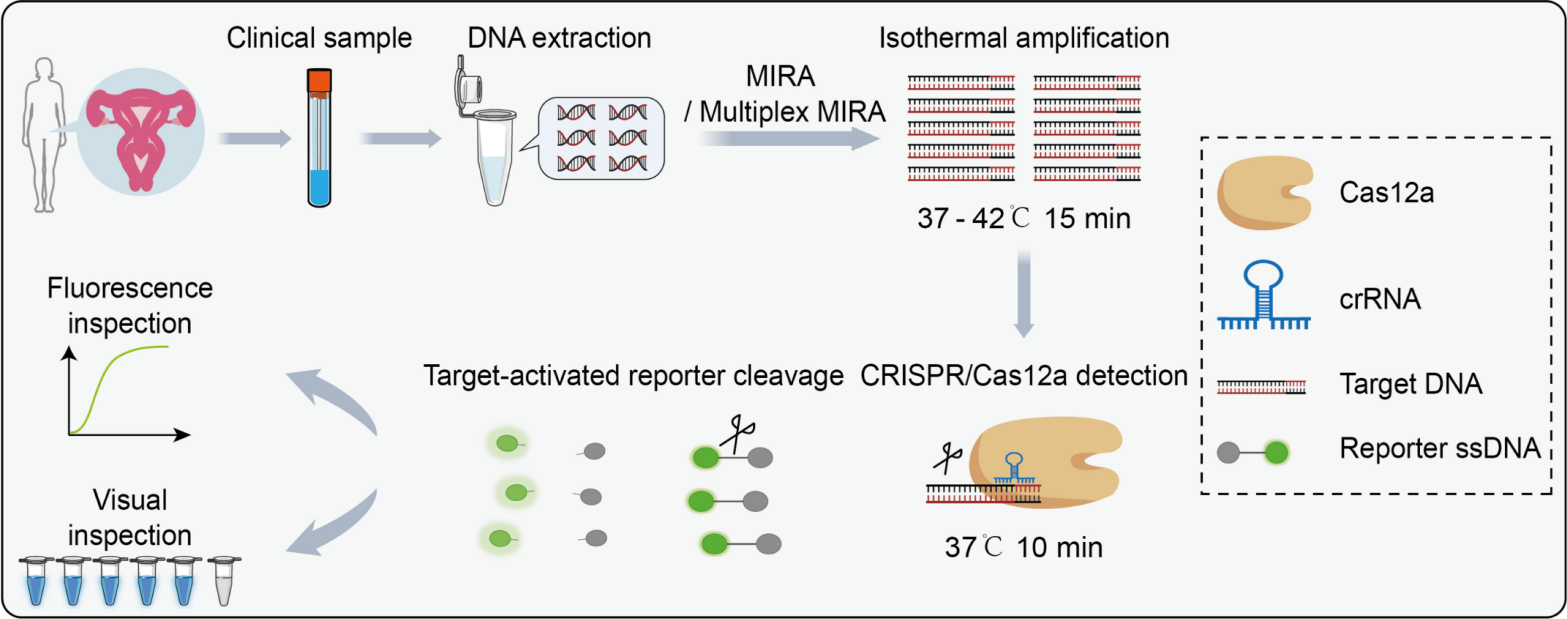
Illustration of the CRISPR/Cas12a-based fluorescent assay for HPV detection in clinical samples. The clinical cervical swab samples are first collected, and genome DNA from the clinical samples is extracted. Then, MIRA or multiplex MIRA is carried out to amplify the 14 HR-HPV genotypes. The amplicons are subsequently detected via the CRISPR/Cas12a system, followed by fluorescence intensity mounting or visualized fluorescence imaging observation.

We developed an efficient HPV DNA detection assay in clinical samples by following the outlined process (Fig. 1). Initially, nucleic acids were obtained from the clinical cervical swab samples. Subsequently, the target gene was amplified using multienzyme isothermal rapid amplification, followed by testing of the amplification products using the fluorescence assay based on CRISPR/Cas12a. When the target amplification products were present, the *trans*-cleavage activity of Cas12a would be triggered, leading to the degradation of the reporter ssDNA and the production of a fluorescent signal. Conversely, the reporter ssDNA would remain intact with no increase in fluorescent signal. Finally, the fluorescent signals could be measured with a microplate reader or visualized by the naked eye after exposure to blue light or ultraviolet.

**Fig 2:**
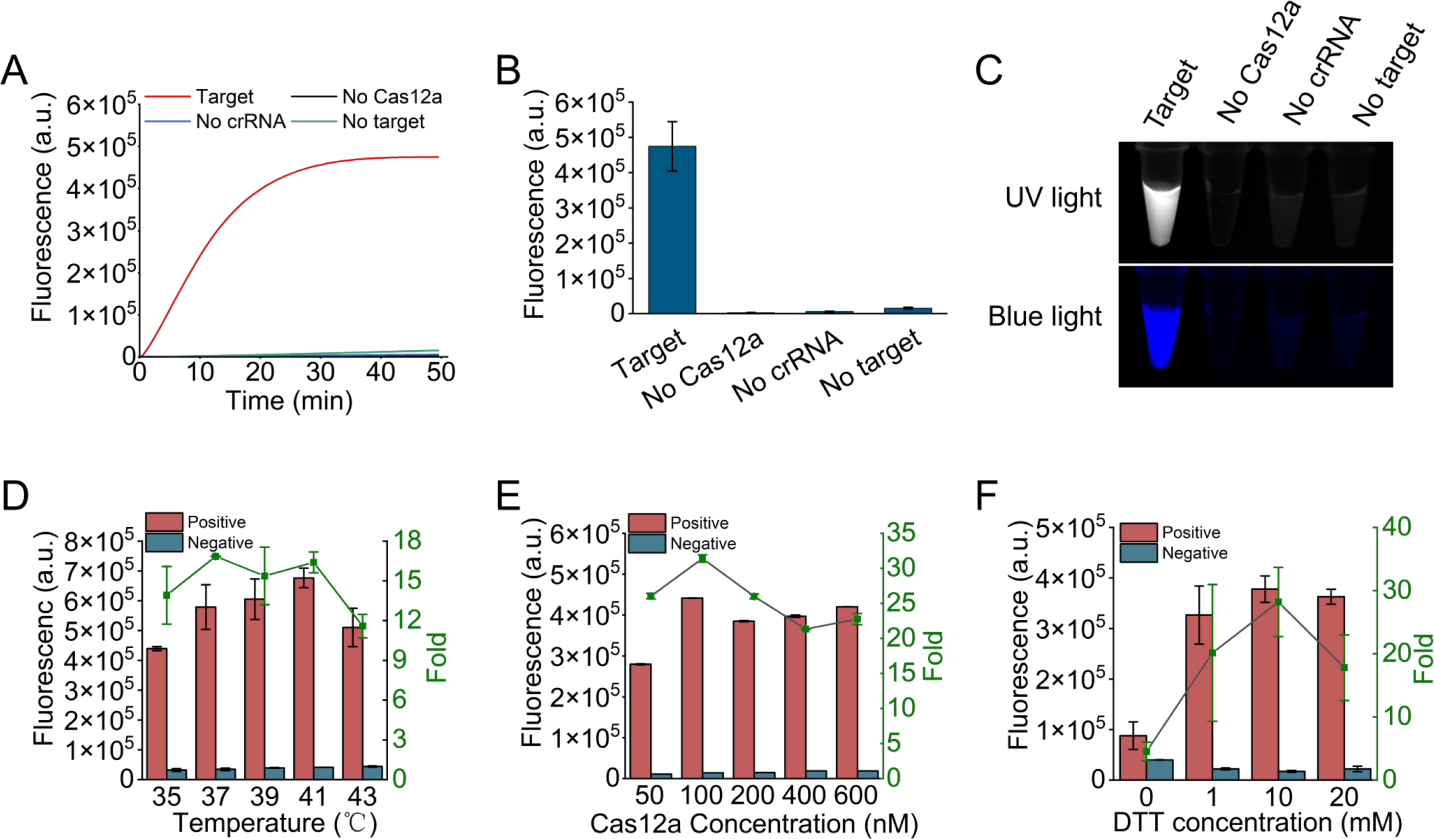
The feasibility and optimization of CRISPR/Cas12a-based fluorescent assay for HPV detection. (**A and B**) The feasibility assessment by real-time (**A**)/30-minute-endpoint (**B**) fluorescence assay to confirm that the *trans*-cleavage activity of Cas12a can be initiated only in the presence of target DNA and correct crRNA. (**C**) The fluorescent images of the samples under a UV light illuminator or blue LED. (**D–F**) The optimization of experimental conditions of CRISPR/Cas12a-based fluorescent assay for HPV detection. The reaction temperature (**D**), concentrations of LbCas12a protein (**E**), and DTT (**F**) were respectively optimized. *n* = 3 technical replicates; error bars represent the mean ± SD.

The feasibility of the proposed MIRA-CRISPR/Cas12a method for nucleic acid detection was evaluated through initial experiments. The HPV L1 gene was selected as the target DNA sequence due to its specific and conservative characteristics. A plasmid DNA containing the L1 gene of HPV16 was utilized as the template DNA for the feasibility assay and amplified using specific primers. The resulting amplicons were then detected using the MIRA-CRISPR/Cas12a assay. Only when the ternary complexes of target DNA, Cas protein, and crRNA were present, the reporter ssDNA was indiscriminately cleaved by the activated Cas12a protein, leading to the enhancement of fluorescent signals (Fig. 2A and B). Conversely, when any of these components were absent, the reported ssDNA remained intact and in a quenched state. Additionally, the fluorescence signal was visually observed under a UV light illuminator or blue LED (Fig. 2C). We further conducted system optimization. For reaction temperature optimization, we found that higher *trans*-cleavage activity occurred at temperatures between 37 and 41℃, with the highest signal-to-background ratio achieved at 37℃ (Fig. 2D). We also tested different concentrations of LbCas12a proteins and found that the highest signal-to-background ratio was observed at 100 nM (Fig. 2E). DTT, a common reductant, can reduce disulfide bonds in proteins, altering their conformation and flexibility. In this study, we compared reaction buffers containing different concentrations of DTT and found that the cleavage activity of Cas12a increased in a DTT concentration-dependent manner, with the activity markedly improved when the DTT concentration reached 10 mM (Fig. 2F).

### Highly sensitive and specific detection of HR-HPV by MIRA-CRISPR/Cas12a method

**Fig 3:**
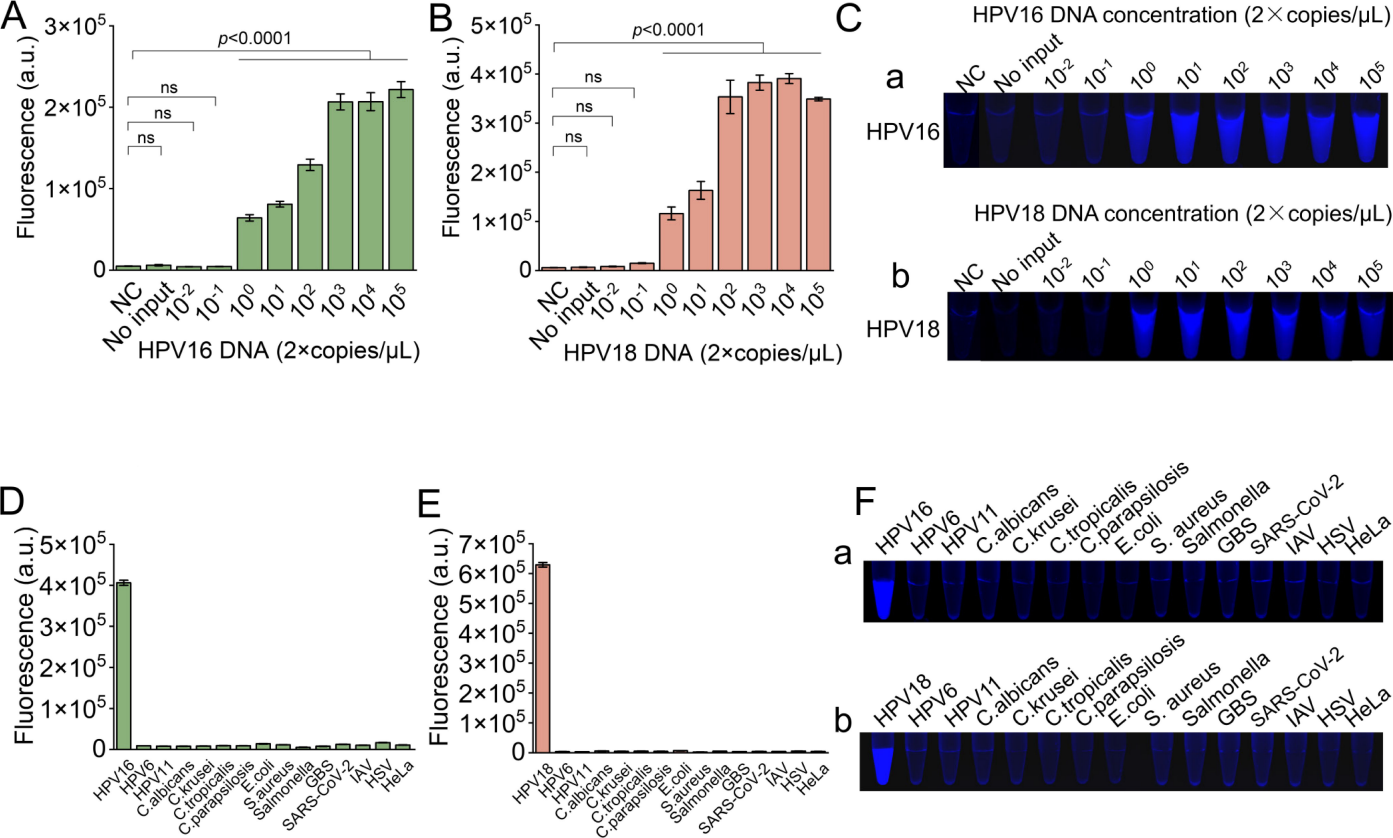
Sensitive and selective detection of HPV by CRISPR/Cas12a-based fluorescent assay. (**A and B**) The sensitivity of CRISPR/Cas12a-based fluorescent assay for HPV16 (**A**) and HPV18 (**B**) detection. The plasmid containing HPV16 or HPV18 L1 gene with a known concentration was gradiently diluted as labeled, which was followed by CRISPR/Cas12a-based fluorescent assay. (**C**) The fluorescent images of HPV16 (a) and HPV18 (b) taken by a blue LED. (**D and E**) The selectivity of CRISPR/Cas12a-based fluorescent assay for HPV16 (**D**) and HPV18 (**E**) detection over other common microorganisms and human cells. (**F**) The fluorescent images of HPV16-selective detection samples (a) and HPV18-selective detection samples (b). *n* = 3 technical replicates; two-tailed Student’s *t*-test; error bars represent the mean ± SD.

HPV16 and HPV18 are the predominant genotypes associated with cervical cancer, accounting for approximately 50% and 20% of cases globally, respectively (27). In this study, HPV16 and HPV18 were selected as representative samples to evaluate the analytical sensitivity and specificity of the method. To assess the sensitivity, we amplified various concentrations of plasmid DNA containing the HPV16 or HPV18 L1 gene using type-specific primers. The amplicons were then detected using the proposed CRISPR/Cas12a-based fluorescent assay. Both HPV16 and HPV18 were efficiently detected with a limit of 2 copies/μL (Fig. 3A and B). Additionally, the intensity of the fluorescent signal increased gradually with the concentration of HPV16 or HPV18 DNA. To simplify the method for field application, a blue LED was utilized to visualize the fluorescent signal (Fig. 3C). To evaluate the analytical specificity of the method, various other microorganisms were tested, including low-risk HPV types 6 and 11, *C. albicans*, *C. krusei*, *C. parapsilosis*, *C. tropicalis*, *E. coli*, *Salmonella*s, group B *Streptococcus*, *S. aureus*, SARS-CoV-2, IAV, HSV, and HeLa cells. The fluorescent signal intensity increased exclusively for HPV16 and HPV18 (Fig. 3D and E), indicating the potential of the assay for selective HPV detection. Similar results were noticed when observing the fluorescent images of the samples, revealing blue fluorescence signals in the presence of HPV16 or HPV18 targets, while no blue fluorescence signals were observed in the interfering groups (Fig. 3F). The detection sensitivity and specificity of the other 12 types of HR-HPV are represented in Fig. S1 and S2.

### Genotyping of HPV by MIRA-CRISPR/Cas12a assay

**Fig 4:**
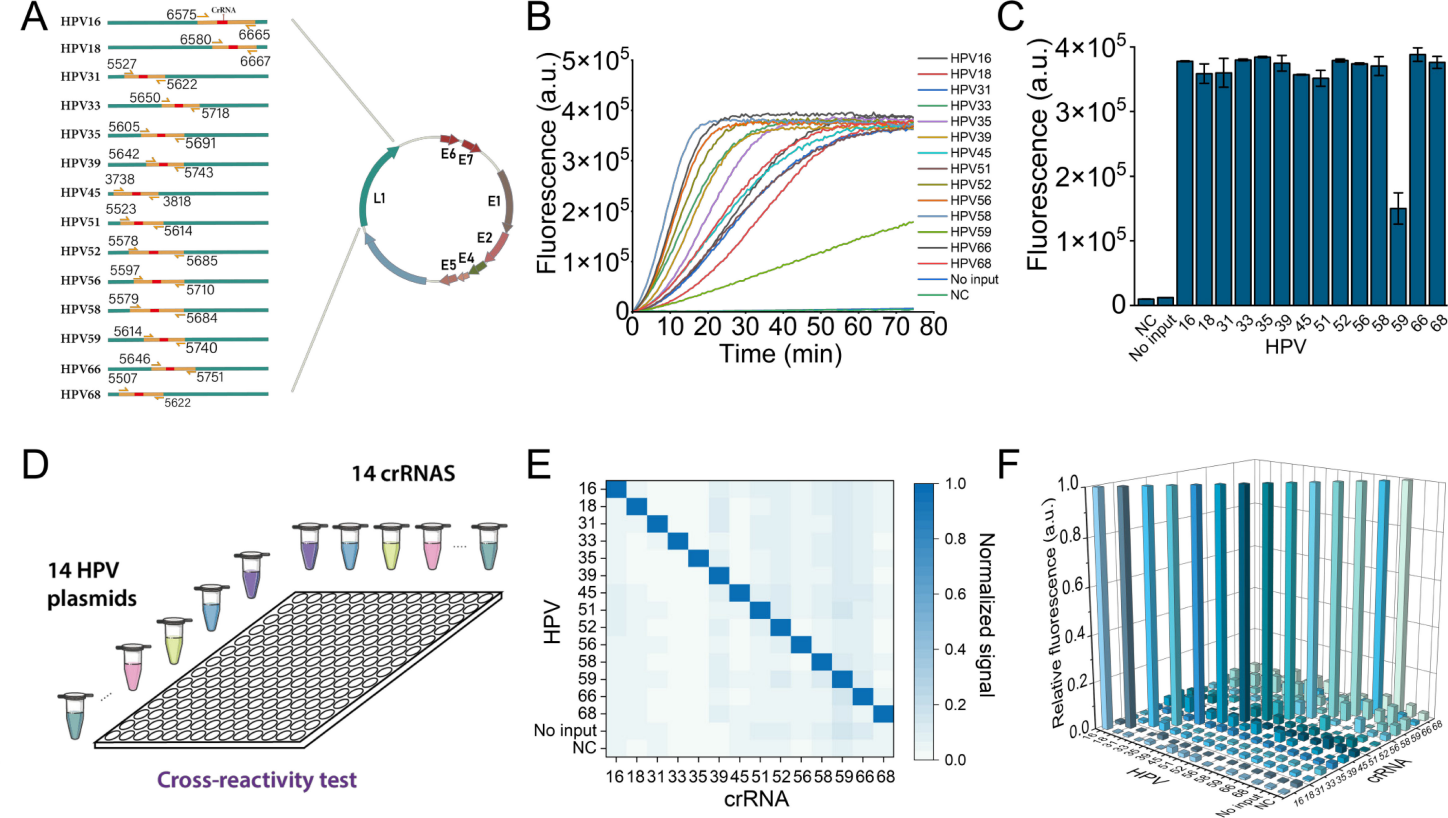
Genotyping detection of HPV by CRISPR/Cas12a-based fluorescent assay. (**A**) Schematic showing the relative position of MIRA primers and crRNA targets located in the conservative L1 region of HPV for the 14 HR-HPV genotypes. (**B and C**) The feasibility analysis of the MIRA-CRISPR/Cas12a-based assay for the 14 HR-HPV genotype detection. The real-time (**B**)/70-minute-endpoint (**C**) CRISPR/Cas12a fluorescence assay for the 14 HR-HPV genotype detection. (**D**) Matrix-based reactivity test of the 14 crRNAs against the 14 HPV genotypes. (**E**) The fluorescence-based test results were demonstrated as a heatmap. (**F**) Quantitative analysis of the reaction of individual crRNAs with the 14 HR-HPV types.

Screening and identifying high-risk HPV infection is crucial to prevent subsequent complications. Herein, we adapted a MIRA-CRISPR/Cas12-based fluorescent assay for detecting 14 high-risk HPV types using the L1 gene to define HPV types. We designed MIRA primers and crRNAs specific to the L1 genes of these HR-HPVs. The locations of amplification primers and crRNAs for each HR-HPV type are shown (Fig. 4A), and their sequences are provided in Table S1. The feasibility of detecting 14 types of HR-HPV using the MIRA-CRISPR/Cas12a assay was tested by using HPV plasmids as templates. The plasmid, lacking the gene insert, served as the negative control group. The fluorescence kinetics curve of the CRISPR/Cas12a reaction displayed strong signals for each HPV type in the presence of the specific target (Fig. 4B), although the cutting efficiency of the crRNA59 group was slightly lower. The fluorescence intensity at 50 minutes was shown (Fig. 4C). After validating the feasibility of the proposed assay for detecting 14 types of HR-HPV, we designed a 14 × 14 matrix test (14 crRNAs × 14 HPV templates; Fig. 4D) to evaluate the capability for genotyping. No widespread cross-activity was observed after the test (Fig. 4E). Similar results were noticed in the individual analysis of the crRNAs (Fig. 4F). Specific MIRA amplification primers and crRNA can distinguish different types of HPV well, suggesting that this method is expected to achieve 14 types of HR-HPV genotyping.

### Multiplex detection of HR-HPV by MIRA-CRISPR/Cas12a assay

**Fig 5:**
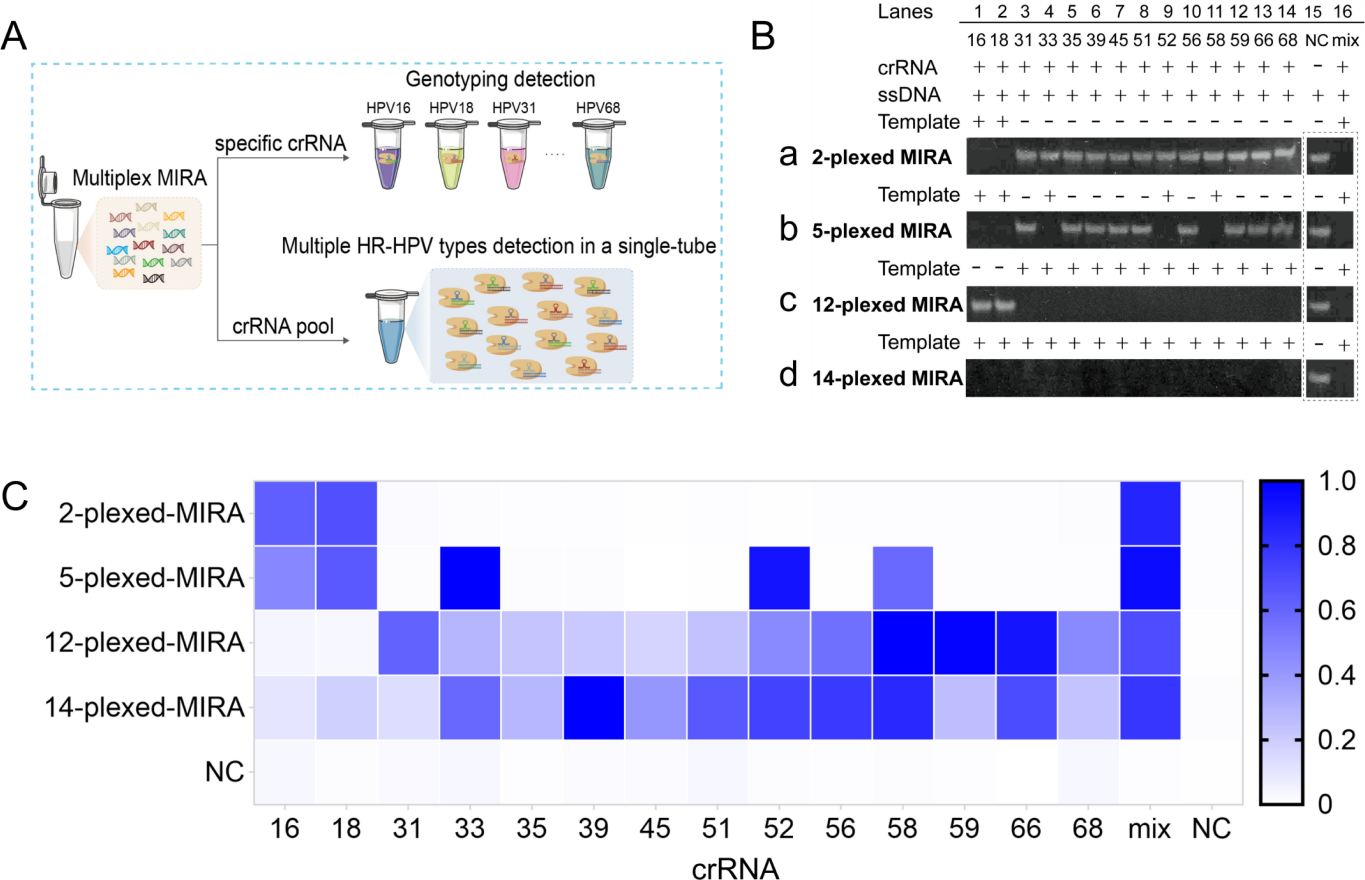
Multiplex detection of HPV by CRISPR/Cas12a assay. (**A**) Schematic diagram of the multiplex detection of HPV by CRISPR/Cas12a assay. HR-HPV genes were amplified by MIRA assay with multiplex HPV primes. The amplification products were detected by CRISPR/Cas12a using individual crRNA or crRNA mixture. (**B**) Denaturing PAGE image representing the cleavage of the reporter ssDNA. Fourteen individual crRNAs or a mixture of crRNAs guided the Cas12a in recognizing the products of 2-, 5-, 12-, and 14-plexed MIRA assays and mediated the cleavage of the reporter ssDNA. (**C**) Heatmap showing the ﬂuorescence-based characterization of the 2-, 5-, 12-, and 14-plexed MIRA products. To be noted, the amplicons used in B and C were from different batches of MIRA assays.

Considering the importance of speed and simplicity in screening for 14 types of HR-HPV, we investigated whether the CRISPR/Cas system could serve as a potent tool for detecting all 14 types of HR-HPV simultaneously using a MIRA primer pool and a crRNA pool (Fig. 5A). To test our hypothesis, we conducted a proof-of-concept assay employing the CRISPR/Cas12a system to test the outputs of the 2-plexed MIRA assay. The targets containing HPV16 and HPV18 L1 genes were amplified using the HPV16 and HPV18 primer pool. Then, the outputs obtained through the 2-plexed MIRA assay were tested by CRISPR/Cas12a assay with the respective crRNA or a crRNA pool. When the Cas12a/crRNA complex recognized the specific amplicon DNA, the Cas12a protein was activated, leading to the nonspecific cleavage of the surrounding ssDNA. Denaturing PAGE was initially employed to examine the degradation of the ssDNA (Fig. 5B (a)). The surrounding ssDNA was cleaved and degraded when the specific crRNA was present (lanes 1 and 2) but remained intact in the absence of the specific crRNA (lanes 3–14). Additionally, it was observed that the surrounding ssDNA was efficiently cleaved when using the HPV16 and HPV18 crRNA pool (lane 16). Furthermore, the 5-, 12-, and 14-plexed MIRA assay were conducted, and the outputs were tested by CRISPR/Cas12a assay. Plasmid without HPV gene insert was used as the negative control. Similarly, denaturing PAGE was initially employed to evaluate the degradation of the surrounding ssDNA (Fig. 5B (b–d)). The surrounding ssDNA was specifically cleaved in the presence of the corresponding crRNA, while it remained intact in the event that the particular crRNA or target DNA was not present. Using the crRNA pool, the 5-, 12-, and 14-plexed MIRA products were also efficiently detected, leading to the cutting of the surrounding ssDNA (lane 16). The original complete denaturing PAGE images are shown in Fig. S3. Next, we evaluated the CRISPR/Cas12a-based detection of 2-, 5-, 12-, and 14-plexed assays using a fluorescence readout. Bright fluorescence signals were observed in the groups containing the specific crRNA or crRNA pool (Fig. 5C), consistent with denaturing PAGE results. These findings confirm the excellent performance of the MIRA-CRISPR/Cas12a method for genotyping and multiplex detection. Specific crRNAs enable accurate genotyping detection of multiplex products, while a mixed pool of crRNAs allows for one-pot screening of multiple high-risk types. This approach simplifies procedures and facilitates rapid primary screening of high-risk HPVs.

### Detection of HPV in clinical specimens using the MIRA-CRISPR/Cas12a method

**Fig 6:**
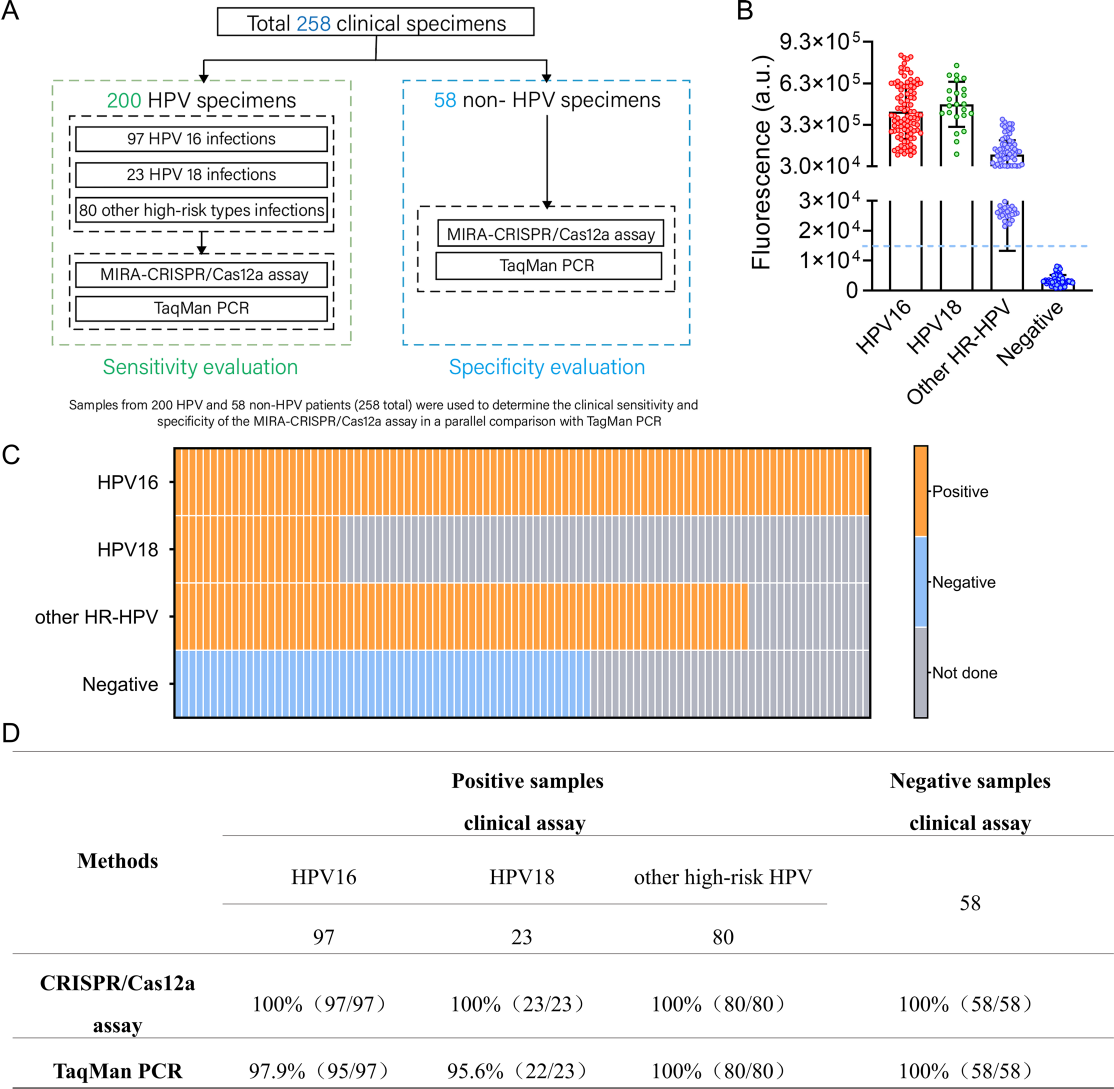
CRISPR/Cas12a assay for HPV detection in clinical specimens. (**A**) Schematic diagram of clinical sample testing. Clinical samples from 200 HR-HPV-infected patients (97 HPV16-infected samples, 23 HPV18-infected samples, and 80 other HR-HPV-infected samples) and 58 healthy persons (without HR-HPV infection) were applied to evaluate the clinical sensitivity and speciﬁcity of the CRISPR/Cas12a assay in a parallel comparison with TaqMan PCR. (**B and C**) Clinical samples were detected by CRISPR/Cas12a-based fluorescence assay. The fluorescence signal values (**B**) and detection results (**C**) of clinical samples were represented, including 97 HPV16-infected cervical swabs, 23 HPV18-infected cervical swabs, 80 other HR-HPV-infected cervical swabs, and 58 uninfected cervical swabs. Colored circles in B indicate clinical cases. The median (interquartile range, IQR) is presented. The blue dashed line indicates the lowest limit of positive signal cutoff threshold. Squares with color in C represent positive signals, while blue squares represent negative results. (**D**) Comparison of the sensitivity and specificity of the MIRA-CRISPR/Cas12a-based method and qPCR.

In order to evaluate the feasibility of clinical detection of this method, 258 clinical specimens were collected and detected, including 97 samples positive with HPV16, 23 samples positive with HPV18, 80 samples positive with other high-risk HPVs (non-16/18 HPV), and 58 negative samples from healthy individuals (Fig. 6A). These clinical samples were first tested and genotyped using a commercial qPCR kit (Cobas HPV test) and confirmed by *in vitro* nucleic acid hybridization assay. In terms of clinical sensitivity evaluation, 200 HR-HPV-positive samples were tested. All HR-HPV-positive samples exhibited higher fluorescence signal intensity (Fig. 6B and C). The clinical detection sensitivity was 100% (97/97 for HPV16, 23/23 for HPV18, and 80/80 for other HR-HPV). In terms of clinical specificity evaluation, 58 HR-HPV-negative samples were tested. All of these samples showed relatively low fluorescence signals, which were below the positive signal cutoff threshold (Fig. 6B). The clinical sample detection specificity was 100%. Given that qPCR is the most frequently used method for detecting HPV nucleic acid, we compared the results of our detection method with those of the commercial qPCR kit. The sensitivity of the MIRA-CRISPR/Cas12a method for HPV detection in clinical samples was markedly higher than that of TaqMan PCR, as demonstrated in Fig. 6D. The MIRA-CRISPR/Cas12a method exhibited 100% sensitivity, whereas the qPCR method demonstrated 97.9% sensitivity (95/97) for HPV16 and 95.6% sensitivity (22/23) for HPV18. These results indicate that the established HPV DNA detection using the MIRA-CRISPR/Cas12a method is highly sensitive, specific, and feasible for clinical sample detection.

## DISCUSSION

Currently, over 200 HPV types have been identified. Among these, 14 HPV types (types 16, 18, 31, 33, 35, 39, 45, 51, 52, 56, 58, 59, 66, and 68) are categorized as high-risk HPV. Each type causes distinct clinical manifestations. For instance, low-risk types such as HPV-6 and HPV-11 are often associated with skin conditions like warts, flat warts, and plantar warts, whereas mucosal high-risk types such as HPV-16 and HPV-18 are linked to more serious conditions including cervical cancer, tonsil cancer, oral cancer, and rectal cancer, among others (3). According to the Centers for Disease Control and Prevention (CDC) information, most sexually active women will contract HPV at least once during their lifetime. Although most infections are subclinical and can be eliminated by the body’s immune system, some HPV infections, particularly HR-HPV infections, can persist for several decades. This can lead to the progression of cervical intraepithelial neoplasia and eventually cervical cancer (28, 29). Early, timely, and regular detection of HPV infections is crucial for preventing and managing cervical cancer. Currently, HPV DNA detection based on qPCR is considered the gold standard for diagnosing HPV infection and has been shown to exhibit higher sensitivity compared to the conventional Pap cytology examination in cervical cancer screening (30). As a result, the majority of commercial HPV diagnostic kits utilize qPCR strategies, including the Cepheid Xpert HPV assay and Roche’s Cobas HPV test (31). However, these techniques necessitate professional personnel, costly equipment, and prolonged reaction time (extraction and testing time typically exceeding 2 hours), rendering them unsuitable for resource-limited settings. Hence, it is essential to devise a faster and more convenient technique to bridge the gap in medical resources.

Besides qPCR-based HPV DNA detection, researchers have developed various isothermal amplification-based methods, including loop-mediated isothermal amplification, recombinase polymerase amplification, strand displacement amplification, rolling circle amplification, and nucleic acid sequence-based amplification (32, 33). However, these methods have shortcomings such as complex primer design, difficulty in multiple and gene typing detection, longer reaction time, and easy occurrence of false positives or false negatives. Recently, detection methods based on CRISPR/Cas systems, including SHERLOCK, HOLMES, and DETECTR, have been increasingly applied to diagnose various pathogens, such as SARS-CoV-2, malaria parasites, pathogenic bacteria, and Mpox (22, 25, 34–37). These methods can also identify single nucleotide variants and monitor medication resistance (38).

Here, we introduced an innovative and effective approach for identifying HR-HPV using the CRISPR/Cas12a-mediated detection system. This approach combined CRISPR/Cas12a with MIRA, which offered double signal amplification and signal recognition specificity, resulting in excellent sensitivity and selectivity. Considering that various factors, such as reaction temperature, Cas12a protein concentration, and reaction buffer, may impact the performance of the CRISPR/Cas12a detection assay (39), the system was optimized. The limit of detection for HPV16/18 was 2 copies/μL with no cross-reactivity. The detection results can also be visualized using UV or blue light. This capability broadens the scope of application by allowing for field-based detection where sophisticated equipment is not available, thereby facilitating rapid and accessible diagnostic procedures in resource-limited settings. Furthermore, accurate genotyping detection of HR-HPV has been achieved by using type-specific amplification primers and crRNA sequences. In addition, rapid and simple detection of all 14 types of HR-HPV is crucial for primary cervical cancer screening, especially when dealing with a large number of samples. In our study, 2-, 5-, 12-, and 14-plexed HR-HPV detection was achieved by combining multiplex amplification with type-specific crRNA or crRNA pool, which exhibited excellent performance in both one-tube detection of all 14 HR-HPV types and two-tube detections (one tube detects HPV16/HPV18, and the other tube detects the remaining 12 types of HR-HPV). This multiplex HPV detection accelerates the screening for HR-HPV infection and conserves significant human and financial resources. To our knowledge, this is the first report to achieve 14-plexed HPV gene amplification and detection. Multiplex isothermal amplification is usually challenged due to the complex primer interference, such as primer dimers and the second structure of primers (40). Previous studies achieved 9-plexed RPA using the optimized RPA primers or 13-plexed RPA using shorter PCR primers (40, 41). In this study, we conducted the multiplex amplification using multienzyme isothermal rapid amplification at a higher temperature of 42℃, rather than 37℃. We hypothesized that elevated temperature could decrease primer interference and improve amplification efficiency. Furthermore, when applied to 258 clinical specimens, our platform demonstrated 100% clinical sensitivity and 100% clinical specificity. Compared to the previously reported CRISPR/Cas-based HPV detection methods that can only detect HPV16/HPV18 or 13 types of HR-HPV (40, 42–46), our platform demonstrated unique advantages, including cost-effectiveness, low complexity, shorter detection time, and the ability to distinguish all the 14 types of high-risk HPV. The comparison of the reported HPV detection with the CRISPR/Cas system is summarized in Table S2. Moreover, our detection platform can be extended to testing other clinically important molecular biology targets in the future, such as tumor markers, bacteria, fungi, or other viruses.

In summary, this platform possesses the benefits of high specificity, high sensitivity, cost-effectiveness, short detection time, independence from expensive equipment, precision genotyping, and multiplicity. We believe that this efficient HPV detection method will rapidly expand the diagnostic screening capabilities and coverage of HPV-infected patients, providing a visual and faster alternative to current PCR-based HPV infection diagnosis and having great potential in point-of-care testing for HR-HPV. It may simplify the process of verifying the treatment of HPV-infected patients and help address global public health issues related to HPV infection.

## AFTER EPUB

[This article was published on 21 November 2024 with an error in the affiliation. The affiliation was corrected in the current version, posted on 06 December 2024.]

## SUPPLEMENTAL MATERIAL

Supplemental figures and tables spectrum.02253-24-s0001.docx Fig. S1 to S3; Tables S1 and S2.

ASM does not own the copyrights to Supplemental Material that may be linked to, or accessed through, an article. The authors have granted ASM a non-exclusive, world-wide license to publish the Supplemental Material files. Please contact the corresponding author directly for reuse.
