# Supplementary material for: Development and evaluation of a CRISPR/Cas12a-based diagnostic test for rapid detection and genotyping of HR-HPV in clinical specimens
Source: Microbiol Spectr. 2024 Nov 21;13(1):e02253-24. doi: 10.1128/spectrum.02253-24 (PMC11705848; doi:10.1128/spectrum.02253-24)
Supplement: Supplemental figures and tables — Fig. S1 to S3; Tables S1 and S2. [file spectrum.02253-24-s0001.docx]

Supplemental material

Development and evaluation of a CRISPR/Cas12a-based diagnostic test for rapid detection and genotyping of HR-HPV in clinical specimens

Lijuan Yin^1#^*, Ziqian Zhao^1#^, Chunhua Wang^2,3#^, Caihong Zhou^1^, Xiuzhen Wu^6^, Baoxue Gao^1^, Liangyuan Wang^1^, Shuli Man^1^, Xinkuan Cheng^1^, Qiankun Wu^7^, Siqi Hu^5^*, Hongxia Fan^4^*, Long Ma^1^*, Hui Xing^2^*, Liang Shen^2^*

^1^ State Key Laboratory of Food Nutrition and Safety, Key Laboratory of Industrial Microbiology, Ministry of Education, Tianjin Key Laboratory of Industry Microbiology, National and Local United Engineering Lab of Metabolic Control Fermentation Technology, China International Science and Technology Cooperation Base of Food Nutrition/Safety and Medicinal Chemistry, College of Biotechnology, Tianjin University of Science & Technology, Tianjin, 300457, China

^2^ Department of Clinical Laboratory, Xiangyang Central Hospital, Affiliated Hospital of Hubei University of Arts and Science, Hubei Province, Xiangyang, China

^3^ Department of Clinical Laboratory, Xiangyang No. 1 People's Hospital, Hubei University of Medicine, Xiangyang, China

^4^ Tianjin Life Science Research Center Tianjin Key Laboratory of Inflammation Biology Collaborative Innovation Center of Tianjin for Medical Epigenetics Department of Pathogen Biology School of Basic Medical Sciences Tianjin Medical University, Tianjin, China.

^5^ Institute of Pediatrics, Faculty of Pediatrics, The Seventh Medical Center of Chinese PLA General Hospital, Beijing, China

^6^ Dynamiker Sub-Center of Beijing Key Laboratory for Mechanisms Research and Precision Diagnosis of Invasive Fungal Disease, Tianjin 300467, China

^7^ Academy of National Food and Strategic Reserves Administration, Beijing, 100037, China

^#^ These authors contributed equally to this work

*Correspondence: shenliang.0829@163.com; yljzhx@tust.edu.cn; [Huixing1969@163.com](mailto:Huixing1969@163.com); malong@tust.edu.cn; fanhongxia@tmu.edu.cn; husiqi_2000@163.com


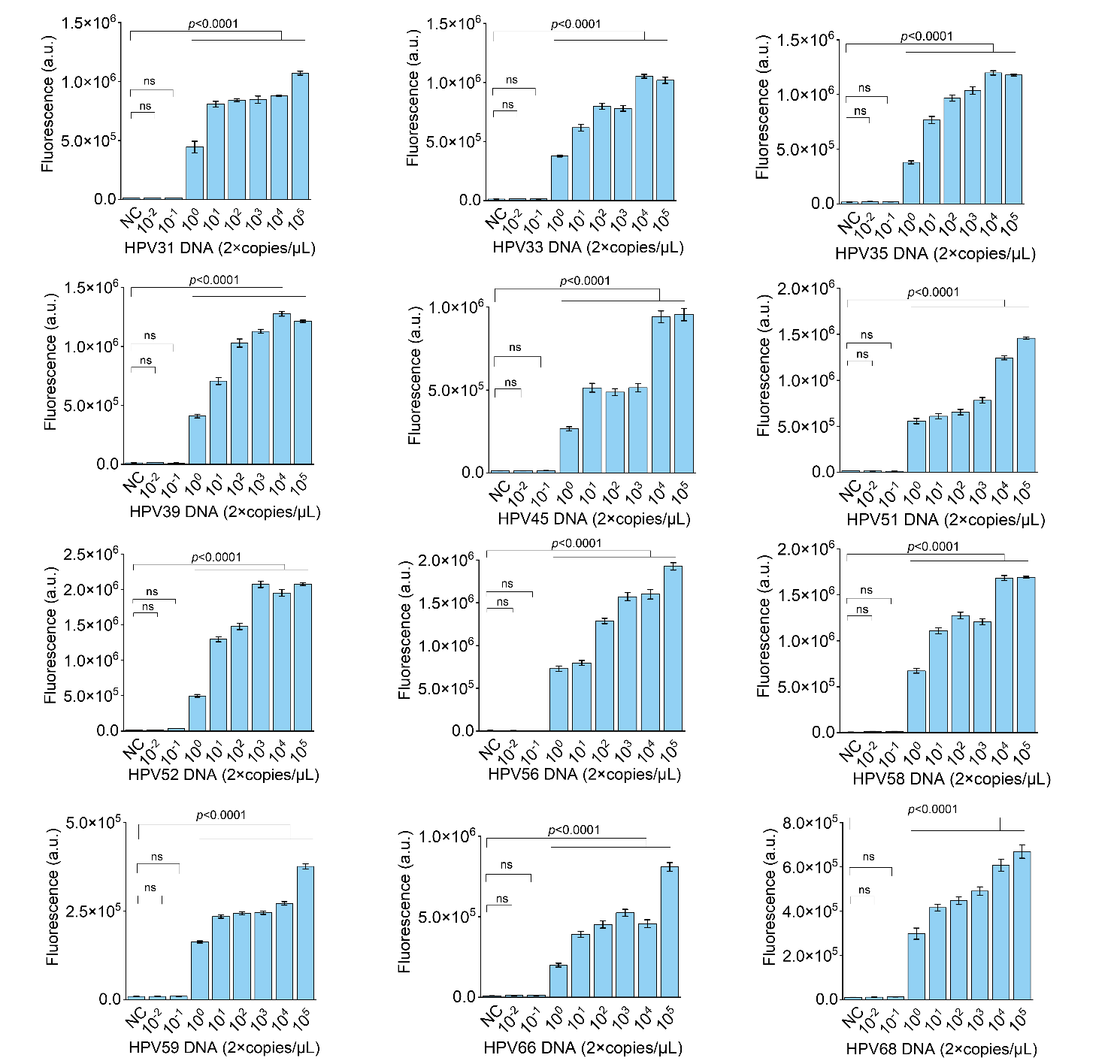


Fig S1 Sensitive detection of other 12 types of HPV by CRISPR/Cas12a-based fluorescent assay.


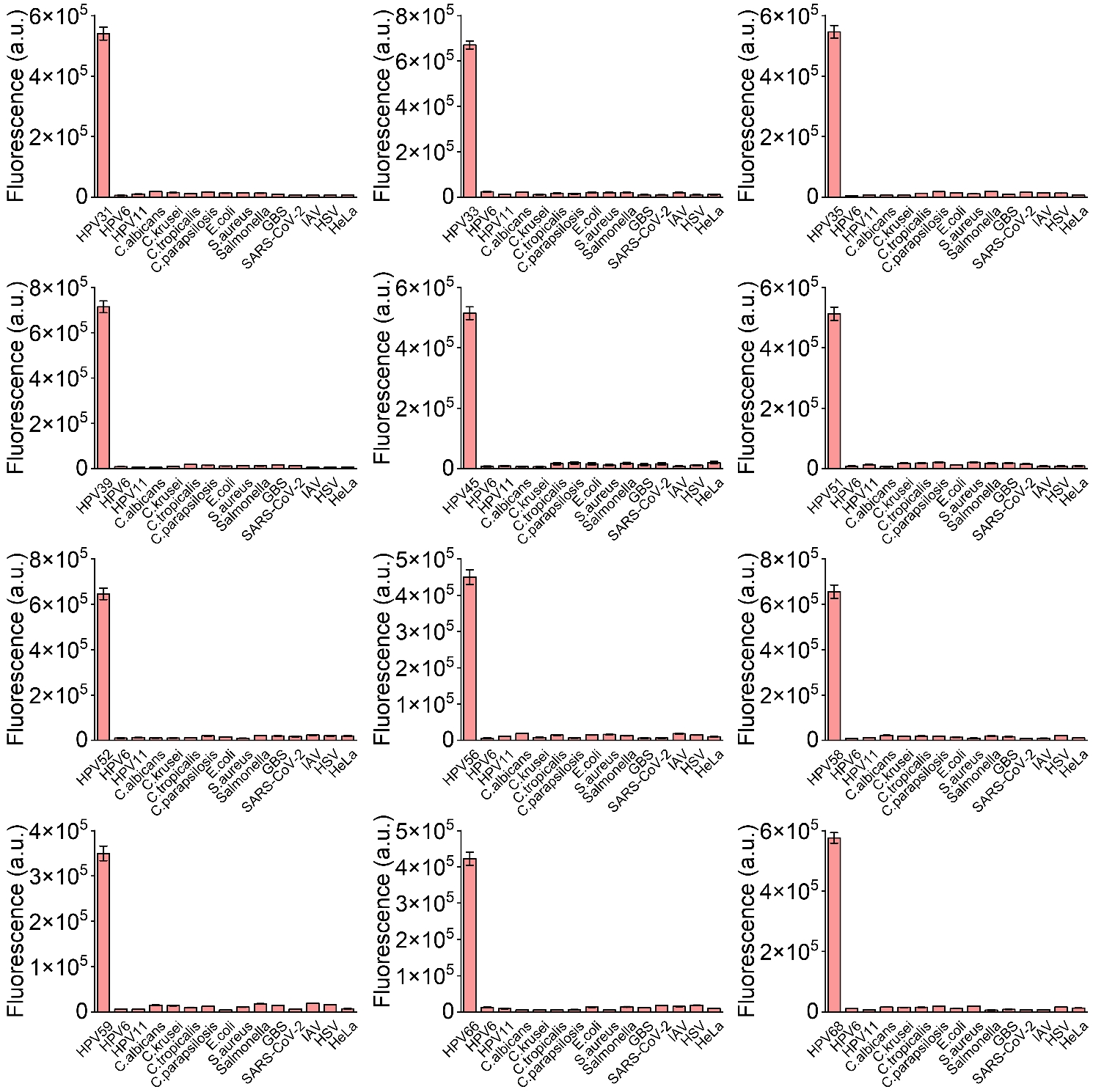


Fig S2 Selective detection of other 12 types of HPV by CRISPR/Cas12a-based fluorescent assay.


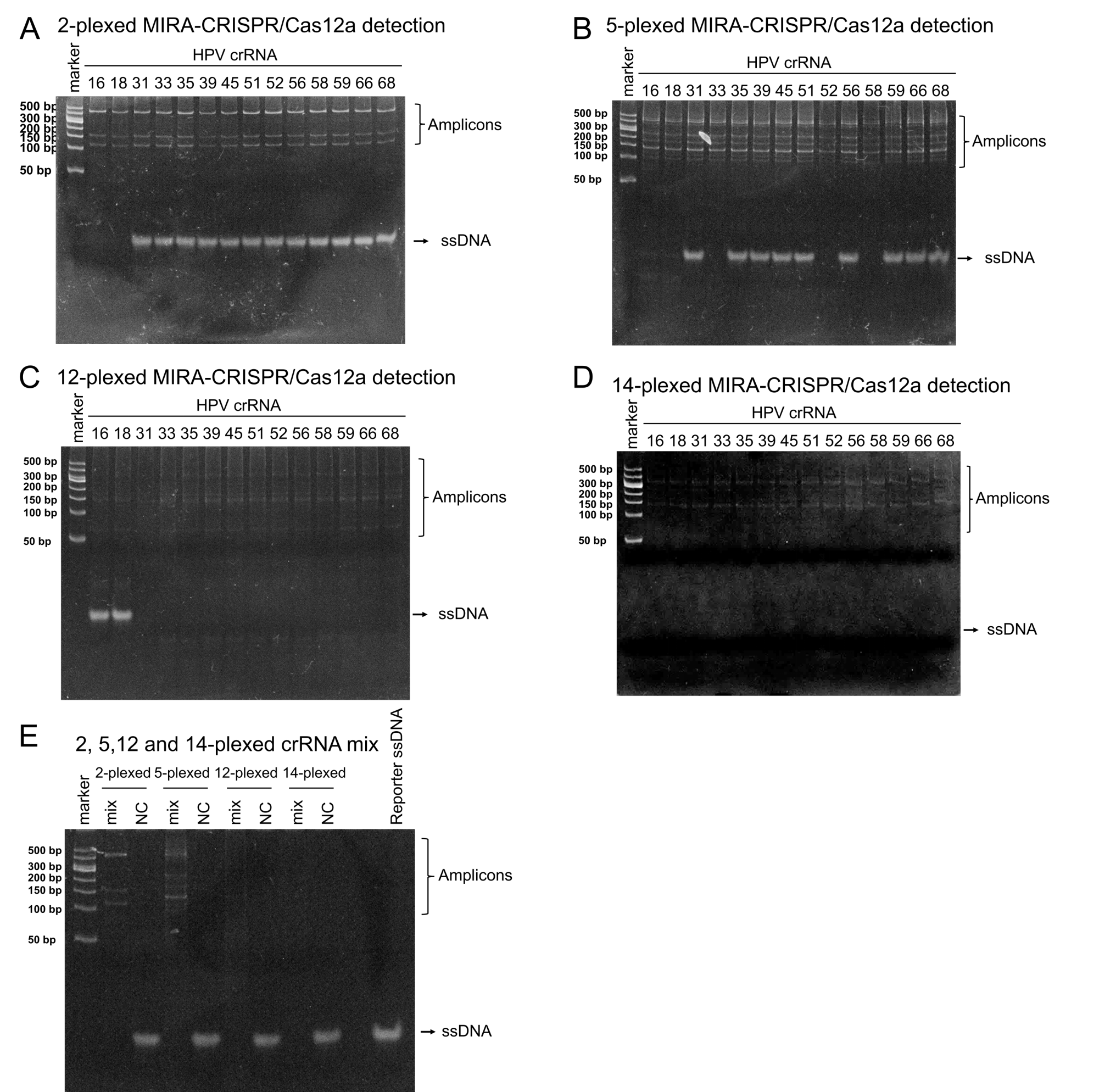


Fig S3 The original complete denaturing PAGE image. (A) PAGE image of 2-plexed MIRA-CRISPR/Cas12a detection. (B) PAGE image of 5-plexed MIRA-CRISPR/Cas12a detection. (C) PAGE image of 12-plexed MIRA-CRISPR/Cas12a detection. (D) PAGE image of 14-plexed MIRA-CRISPR/Cas12a detection. (E) PAGE image of 2-plexed, 5-plexed, 12-plexed, and 14-plexed amplicons detection with corresponding crRNA pool.

.

Table S1. Primers and crRNA sequences used in the study

| Primer ID | Target gene | Sequence (5’ to 3’)^a^ | Application in  this study | Reference |
| --- | --- | --- | --- | --- |
| HPV16 Forward | HPV16 L1 | cacctagtggttctatggttacctctgatgccca | MIRA | This study |
| HPV16 Reverse | HPV16 L1 | tatgtagtttctgaagtagatatggcagcacataa | MIRA | This study |
| HPV18 Forward | HPV18 L1 | gcataatcaattatttgttactgtggtagatacc | MIRA | This study |
| HPV18 Reverse | HPV18 L1 | gctatactgcttaaatttggtagcatcatattgc | MIRA | This study |
| HPV31 Forward | HPV31 L1 | atgtctctgtggcggcctagcgaggctactgtcta | MIRA | This study |
| HPV31 Reverse | HPV31 L1 | taagcagcctagcactgcctgcgtgataatatatg | MIRA | This study |
| HPV33 Forward | HPV33 L1 | ctaaagttgtcagcactgatgaatatgtgtctcgc | MIRA | This study |
| HPV33 Reverse | HPV33 L1 | aatagaaaaatatggatggccaacagcaagaag | MIRA | This study |
| HPV35 Forward | HPV35 L1 | tctgtggcggtctaacgaagccactgtctacct | MIRA | This study |
| HPV35 Reverse | HPV35 L1 | gcctagaactgcctgcatgatagtagatgtttg | MIRA | This study |
| HPV39 Forward | HPV39 L1 | atggctctgtggcggtctagtgacagcatggtg | MIRA | This study |
| HPV39 Reverse | HPV39 L1 | actgttaataatctagagctgccagcataataa | MIRA | This study |
| HPV45 Forward | HPV45 L1 | tattattttcctaaaaaacgtaaacgtattccc | MIRA | This study |
| HPV45 Reverse | HPV45 L1 | tgacaactctggccacagaaggtggtggaagat | MIRA | This study |
| HPV51 Forward | HPV51 L1 | atggcattgtggcgcactaatgacagcaaggtg | MIRA | This study |
| HPV51 Reverse | HPV51 L1 | gtctggaactgcctgcatagtaatatatgccgg | MIRA | This study |
| HPV52 Forward | HPV52 L1 | attttacatcctagttattttttactacgtcgc | MIRA | This study |
| HPV52 Reverse | HPV52 L1 | catcagtgcttacaaccttagagacaggtacag | MIRA | This study |
| HPV56 Forward | HPV56 L1 | atggcgacgtggcggcctagtgaaaataaggtg | MIRA | This study |
| HPV56 Reverse | HPV56 L1 | tagggatgtcctacggcaagcaatcgtgaactg | MIRA | This study |
| HPV58 Forward | HPV58 L1 | tgttgcaccctagctattttattttgcgtcgca | MIRA | This study |
| HPV58 Reverse | HPV58 L1 | atcagtgcttacaaccttagacacaggcacagg | MIRA | This study |
| HPV59 Forward | HPV59 L1 | tggcgttctagtgacaacaaggtgtatctacc | MIRA | This study |
| HPV59 Reverse | HPV59 L1 | accacctttaggtactttaaaatatggatgtcc | MIRA | This study |
| HPV66 Forward | HPV66 L1 | atggcgatgtggcggcctagtgacaataaggtg | MIRA | This study |
| HPV66 Reverse | HPV66 L1 | gccaacagcaagcaacctagagctacctgcatg | MIRA | This study |
| HPV68 Forward | HPV68 L1 | atggcattgtggcgctctagcgacaacatggtg | MIRA | This study |
| HPV68 Reverse | HPV68 L1 | aatatggatggcctacagttaataacctagatg | MIRA | This study |
| HPV16 crRNA | HPV16 L1 | cgtgtagtatcaacaacagtaaATCTACACTTAGTAGAAATTA | CRISPR/Cas12a assay | This study |
| HPV18 crRNA | HPV18 L1 | tgtgtagaagcacatattgtATCTACACTTAGTAGAAATTA | CRISPR/Cas12a assay | This study |
| HPV31 crRNA | HPV31 L1 | gttacatattcatccgtgcttaATCTACACTTAGTAGAAATTA | CRISPR/Cas12a assay | This study |
| HPV33 crRNA | HPV33 L1 | tctggaactaccagcataataaATCTACACTTAGTAGAAATTA | CRISPR/Cas12a assay | This study |
| HPV35 crRNA | HPV35 L1 | actgatgaatatgtaacacgcaATCTACACTTAGTAGAAATTA | CRISPR/Cas12a assay | This study |
| HPV39 crRNA | HPV39 L1 | ccttcgccacagaaggtggaggATCTACACTTAGTAGAAATTA | CRISPR/Cas12a assay | This study |
| HPV45 crRNA | HPV45 L1 | ccgtactgtcactaggccgccaATCTACACTTAGTAGAAATTA | CRISPR/Cas12a assay | This study |
| HPV51 crRNA | HPV51 L1 | ttcgagacacaggtgcaggtggATCTACACTTAGTAGAAATTA | CRISPR/Cas12a assay | This study |
| HPV52 crRNA | HPV52 L1 | actacgtcgcaggcgtaaacgtATCTACACTTAGTAGAAATTA | CRISPR/Cas12a assay | This study |
| HPV56 crRNA | HPV56 L1 | ggaatccgttgccacaacctttATCTACACTTAGTAGAAATTA | CRISPR/Cas12a assay | This study |
| HPV58 crRNA | HPV58 L1 | ttacgtctgcgacgcaaaatATCTACACTTAGTAGAAATTA | CRISPR/Cas12a assay | This study |
| HPV59 crRNA | HPV59 L1 | gtctggaactgcctgcgtggtaATCTACACTTAGTAGAAATTA | CRISPR/Cas12a assay | This study |
| HPV66 crRNA | HPV66 L1 | tgtatccgttgccacaacctttATCTACACTTAGTAGAAATTA | CRISPR/Cas12a assay | This study |
| HPV68 crRNA | HPV68 L1 | ccttcgccactgaggggggaggATCTACACTTAGTAGAAATTA | CRISPR/Cas12a assay | This study |

^a^ The capitalized nucleic acid bases denote the crRNA stem-loop sequences.

Table S2. Comparison of our proposed MIRA-CRISPR/Cas12a assay with others reported methods for HPV detection.

| Method | Type | Effector | LOD | Visualization | Assay time | Multiplex | Quantification | POCT | Refs |
| --- | --- | --- | --- | --- | --- | --- | --- | --- | --- |
| DNA tetrahedron-based CRISPR bioassay | HPV (16,18,52) | Cas12a | 218 fM | No | - | Yes | Yes | No | (1) |
| CRISPR/Cas12a | HPV16 | Cas12a | 8.86 fM | No | ＜100 min | No | Yes | No | (2) |
| Electric Field-Enhanced Electrochemical CRISPR Biosensor | HPV16 | Cas12a | 1 pM | No | - | No | No | Yes | (3) |
| CRISPR/Cas12a | HPV16 | Cas12a | 0.32 pM | No | ＜2 h | Yes | Yes | No | (4) |
| NEXT CRISPR | HPV16 | Cas12a | 0.17 fM | Yes | 30 min | No | No | Yes | (5) |
| G-CRISPR | HPV (16,18) | Cas12a | 0.1 aM | Yes | 35 min | No | No | Yes | (6) |
| CRISPR/Cas12a-mediated AuNPs aggregation-based surface-enhanced Raman scattering platform | HPV (16,18) | Cas12a | 6.72 pM | No | 40 min | No | No | Yes | (7) |
| ctPCR3.0 | HPV (16, 18, 33,  35, 45, 51, 52, 56, 58, 59) | Cas9 | 180 copies | No | ＜2 h | No | Yes | No | (8) |
| CRISPR-CID | HPV (16,18) | Cas12a | 1 copy | Yes | - | No | Yes | No | (9) |
| CRISPR‑Cas12a technology | HPV (16, 18, 31, 33, 35, 39, 45, 51, 52, 56, 58, 59, 68) | Cas12a | 500 copies | No | 35 min | Yes | No | No | (10) |
| CIALFB | HPV (16,18) | Cas12a | 3.1 attomoles  (∼1.8 copies) | No | < 1 h | No | No | Yes | (11) |
| CADD | HPV (16, 18, 31, 33, 35, 39, 45, 51, 52, 56, 58, 59, 66,68,73) | Cas9 | 10 fM | Yes | 30 min | No | No | Yes | (12) |
| CRISPR/Cas9 | HPV(16,18,33) | Cas9 | 0.025 pM | Yes | 2 h | Yes | Yes | No | (13) |
| ctPCR4.0 | HPV (16, 18, 33, 35, 45, 51, 52, 56, 58, 59) | Cas9 | - | No | 2-3 h | Yes | Yes | No | (14) |
| CRISPR test | HPV (16,18) | Cas12a | 0.24 fM | Yes | - | No | No | Yes | (15) |
| M3-CRISPR | HPV (16,18) | Cas12a | 10 ^–18^ M | Yes | 30 min | Yes | No | Yes | (16) |
| M-D3 | HPV (16,18) | Cas12a | 10 ^–18^ M | Yes | 30 min | Yes | Yes | Yes | (17) |
| CRISPRCas13a/Cas12a dual-channel system | HPV (16,18) | Cas13a/Cas12a | 10^0^ copy/μL | Yes | 1 h | Yes | No | Yes | (18) |
| MiCaR | HPV (6,11,16,18,31,33,45,52,58) | Cas12a | 0.26 attomole | Yes | 40 min | Yes | No | Yes | (19) |
| CRISPR/dCas9-mediated surface enhanced raman scattering strategy | HPV16 | Cas9 | 30–190 ng | No | - | No | Yes | No | (20) |
| CRISPR-Cas12a-driven MXene-PEDOT | HPV16 | Cas12a | 534 pM | Yes | - | No | No | No | (21) |
| CRISPR/Cas12a assay | HPV16 | Cas12a | 100 fm | No | - | No | No | Yes | (22) |
| CARP | HPV (16,18) | Cas9 | 0.02 ng | No | 3 h | No | Yes | No | (23) |
| CRISPR-Mediated Detection | HPV (16,18) | Cas12a | 1 aM-100 pM | No | 20 min | Yes | Yes | Yes | (24) |
| R-CASTI | HPV (16,18) | Cas12a | 1 aM | No | 80 min | Yes | Yes | Yes | (25) |
| CRICED | HPV (16,18) | Cas12a | 17 pM | Yes | - | No | No | No | (26) |
| Homogeneous electrochemical biosensor based on CRISPR-Cas12a | HPV16 | Cas12a | 3.22 pM | No | 50 min | No | Yes | No | (27) |
| ctPCR | HPV (16,18) | Cas9 | 1 copy/μL | No | 3-4 h | No | No | No | (28) |
| PddCas | HPV18 | Cas12a | 100 aM | No | ＜30 min | No | Yes | Yes | (29) |
| Size-controlled engineering photoelectrochemical biosensor based on CRISPR-Cas12a | HPV16 | Cas12a | 1 pM | No | - | No | Yes | No | (30) |
| CRISPR-Cas12a-derived photoelectrochemical biosensor | HPV16 | Cas12a | 1.2 pM | No | - | No | Yes | Yes | (31) |
| CasDOS | HPV (16,18) | Cas12a | 16.6 aM | No | 30 min | No | No | Yes | (32) |
| Electrochemical Strategy for Viral Detection | HPV (16,18) | Cas12a | 10^4^ copies/reaction | No | 1 h | No | Yes | Yes | (33) |
| Interactive dual-readout differential lateral flow biosensor | HPV (16,18) | Cas12a | 0.21 pM for HPV-18 and 42.92 pM for HPV-16 | Yes | 30 min | Yes | Yes | Yes | (34) |
| CRISPR/Cas12a on Surface Binding of Polymer Dots | HPV16 | Cas12a | 2.3 fM | No | - | No | Yes | No | (35) |
| CRISPR/Cas12a-mediated liposome-amplified strategy | HPV16 | Cas12a | 1.6 pM | No | - | No | Yes | No | (36) |
| MIRA-CRISPR/Cas12a method | HPV (16, 18, 31, 33, 35, 39, 45, 51, 52, 56, 58, 59, 66, 68) | Cas12a | 2 copies/μL | Yes | 30 min | Yes | Yes | Yes | Our method |

References

1. Zhan X, Zhou J, Jiang Y, An P, Luo B, Lan F, Ying B, Wu Y. 2023. DNA tetrahedron-based CRISPR bioassay for treble-self-amplified and multiplex HPV-DNA detection with elemental tagging. *Biosens Bioelectron* 229:115229.

2. Yu L, Peng Y, Sheng M, Wang Q, Huang J, Yang X. 2023. Sensitive and amplification-free electrochemiluminescence biosensor for HPV-16 detection based on CRISPR/Cas12a and DNA tetrahedron nanostructures. *ACS Sens* 8:2852-2858.

3. Li Z, Ding X, Yin K, Xu Z, Cooper K, Liu C. 2021. Electric field-enhanced electrochemical CRISPR biosensor for DNA detection. *Biosens Bioelectron* 192:113498.

4. Zhao KR, Wang L, Liu PF, Hang XM, Wang HY, Ye SY, Liu ZJ, Liang GX. 2021. A signal-switchable electrochemiluminescence biosensor based on the integration of spherical nucleic acid and CRISPR/Cas12a for multiplex detection of HIV/HPV DNAs. *Sensor Actuat B-Chem* 346:130485.

5. Ganbaatar U, Liu CC. 2022. NEXT CRISPR: An enhanced CRISPR-based nucleic acid biosensing platform using extended crRNA. *Sensor Actuat B-Chem* 369:132296.

6. Li T, Hu R, Xia J, Xu Z, Chen D, Xi J, Liu BF, Zhu J, Li Y, Yang Y, Liu M. 2021. G-triplex: A new type of CRISPR-Cas12a reporter enabling highly sensitive nucleic acid detection. *Biosens Bioelectron* 187:113292.

7. Su AL, Liu Y, Cao XM, Xu WQ, Liang CY, Xu SP. 2022. A universal CRISPR/Cas12a-mediated AuNPs aggregation-based surface-enhanced Raman scattering (CRISPR/Cas-SERS) platform for virus gene detection. *Sensor Actuat B-Chem* 369:132295.

8. Zhang B, Xia Q, Wang Q, Xia X, Wang J. 2018. Detecting and typing target DNA with a novel CRISPR-typing PCR (ctPCR) technique. *Anal Biochem* 561-562:37-46.

9. Ke X, Ou Y, Lin Y, Hu T. 2022. Enhanced chemiluminescence imaging sensor for ultrasensitive detection of nucleic acids based on HCR-CRISPR/Cas12a. *Biosens Bioelectron* 212:114428.

10. Gong J, Zhang G, Wang W, Liang L, Li Q, Liu M, Xue L, Tang G. 2021. A simple and rapid diagnostic method for 13 types of high-risk human papillomavirus (HR-HPV) detection using CRISPR-Cas12a technology. *Sci Rep* 11:12800.

11. Mukama O, Yuan T, He ZX, Li ZY, Habimana JD, Hussain M, Li W, Yi ZJ, Liang QX, Zeng LW. 2020. A high fidelity CRISPR/Cas12a based lateral flow biosensor for the detection of HPV16 and HPV18. *Sensor Actuat B-Chem* 316:128119.

12. Xu X, Luo T, Gao J, Lin N, Li W, Xia X, Wang J. 2020. CRISPR-assisted DNA detection: A novel dCas9-based DNA detection technique. *CRISPR J* 3:487-502.

13. Zhang DG, Cai LJ, Wei XW, Wang YT, Shang LR, Sun LY, Zhao YJ. 2021. Multiplexed CRISPR/Cas9 quantifications based on bioinspired photonic barcodes. *Nano Today* 40:101268.

14. Gao J, Wu L, Yang D, Gong W, Wang J. 2021. A one-pot CRISPR/Cas9-typing PCR for DNA detection and genotyping. *J Mol Diagn* 23:46-60.

15. Tsou JH, Leng Q, Jiang F. 2019. A CRISPR test for detection of circulating nuclei acids. *Transl Oncol* 12:1566-1573.

16. Zhou H, Xu Z, He L, Wang Z, Zhang T, Hu T, Huang F, Chen D, Li Y, Yang Y, Huang X. 2023. Coupling CRISPR/Cas12a and recombinase polymerase amplification on a stand-alone microfluidics platform for fast and parallel nucleic acid detection. *Anal Chem* 95:3379-3389.

17. Zhao Y, Chen D, Xu Z, Li T, Zhu J, Hu R, Xu G, Li Y, Yang Y, Liu M. 2023. Integrating CRISPR-Cas12a into a microfluidic dual-droplet device enables simultaneous detection of HPV16 and HPV18. *Anal Chem* 95:3476-3485.

18. Zheng X, Li Y, Yuan M, Shen Y, Chen S, Duan G. 2022. Rapid detection of HPV16/18 based on a CRISPR-Cas13a/Cas12a dual-channel system. *Anal Methods* 14:5065-5075.

19. Xu Z, Chen D, Li T, Yan J, Zhu J, He T, Hu R, Li Y, Yang Y, Liu M. 2022. Microfluidic space coding for multiplexed nucleic acid detection via CRISPR-Cas12a and recombinase polymerase amplification. *Nat Commun* 13:6480.

20. Su A, Liu Y, Cao X, Zhao J, Xu W, Liang C, Li P, Xu S. 2023. Direct virus gene detection: A CRISPR/dCas9-mediated surface-enhanced Raman scattering strategy with enzyme-catalyzed signal amplification. *Anal Chem* 95:5927-5936.

21. Zeng RJ, Wang WJ, Chen MM, Wan Q, Wang CC, Knopp D, Tang DP. 2021. CRISPR-Cas12a-driven MXene-PEDOT:PSS piezoresistive wireless biosensor. *Nano Energy* 82:105711.

22. Su J, Ke Y, Maboyi N, Zhi X, Yan S, Li F, Zhao B, Jia X, Song S, Ding X. 2021. CRISPR/Cas12a powered DNA framework-supported electrochemical biosensing platform for ultrasensitive nucleic acid analysis. *Small Methods* 5:e2100935.

23. Zhang B, Wang Q, Xu X, Xia Q, Long F, Li W, Shui Y, Xia X, Wang J. 2018. Detection of target DNA with a novel Cas9/sgRNAs-associated reverse PCR (CARP) technique. *Anal Bioanal Chem* 410:2889-2900.

24. Choi JH, Shin M, Yang L, Conley B, Yoon J, Lee SN, Lee KB, Choi JW. 2021. Clustered regularly interspaced short palindromic repeats-mediated amplification-free detection of viral DNAs using surface-enhanced Raman spectroscopy-active nanoarray. *ACS Nano* 15:13475-13485.

25. Han J, Shin J, Lee ES, Cha BS, Kim S, Jang Y, Kim S, Park KS. 2023. Cas12a/blocker DNA-based multiplex nucleic acid detection system for diagnosis of high-risk human papillomavirus infection. *Biosens Bioelectron* 232:115323.

26. Hu T, Ke X, Ou Y, Lin Y. 2022. CRISPR/Cas12a-Triggered chemiluminescence enhancement biosensor for sensitive detection of nucleic acids by introducing a tyramide signal amplification strategy. *Anal Chem* 94:8506-8513.

27. Liu J, Wan Q, Zeng R, Tang D. 2021. An ultrasensitive homogeneous electrochemical biosensor based on CRISPR-Cas12a. *Anal Methods* 13:3227-3232.

28. Wang Q, Zhang B, Xu X, Long F, Wang J. 2018. CRISPR-typing PCR (ctPCR), a new Cas9-based DNA detection method. *Sci Rep* 8:14126.

29. Xue Y, Luo X, Xu W, Wang K, Wu M, Chen L, Yang G, Ma K, Yao M, Zhou Q, Lv Q, Li X, Zhou J, Wang J. 2023. PddCas: A polydisperse droplet digital CRISPR/Cas-based assay for the rapid and ultrasensitive amplification-free detection of viral DNA/RNA. *Anal Chem* 95:966-975.

30. Li Y, Zeng R, Wang W, Xu J, Gong H, Li L, Li M, Tang D. 2022. Size-controlled engineering photoelectrochemical biosensor for human papillomavirus-16 based on CRISPR-Cas12a-induced disassembly of Z-scheme heterojunctions. *ACS Sens* 7:1593-1601.

31. Zeng R, Gong H, Li Y, Li Y, Lin W, Tang D, Knopp D. 2022. CRISPR-Cas12a-derived photoelectrochemical biosensor for point-of-care diagnosis of nucleic acid. *Anal Chem* 94:7442-7448.

32. Zeng Q, Zhou M, Hu Z, Deng W, Li Z, Wu L, Liang D. 2023. Rapid and sensitive Cas12a-based one-step nucleic acid detection with ssDNA-modified crRNA. *Anal Chim Acta* 1276:341622.

33. Zamani M, Robson JM, Fan A, Bono MS, Jr., Furst AL, Klapperich CM. 2021. Electrochemical Strategy for Low-Cost Viral Detection. *ACS Cent Sci* 7:963-972.

34. Li L, Tian H, Wang G, Ren S, Ma T, Wang Y, Ge S, Zhang Y, Yu J. 2023. Ready-to-use interactive dual-readout differential lateral flow biosensor for two genotypes of human papillomavirus. *Biosens Bioelectron* 228:115224.

35. Li L, Yu S, Wu J, Ju H. 2023. Regulation of target-activated CRISPR/Cas12a on surface binding of polymer dots for sensitive electrochemiluminescence DNA analysis. *Anal Chem* 95:7396-7402.

36. Gong H, Wu Y, Zeng R, Zeng Y, Liu X, Tang D. 2021. CRISPR/Cas12a-mediated liposome-amplified strategy for the photoelectrochemical detection of nucleic acid. *Chem Commun (Camb)* 57:8977-8980.
